# Tobacco Control Measures to Reduce Socioeconomic Inequality in Smoking: The Necessity, Time-Course Perspective, and Future Implications

**DOI:** 10.2188/jea.JE20160206

**Published:** 2018-04-05

**Authors:** Takahiro Tabuchi, Hiroyasu Iso, Eric Brunner

**Affiliations:** 1 Cancer Control Center, Osaka International Cancer Institute, Osaka, Japan; 2 Public Health, Department of Social Medicine, Osaka University Graduate School of Medicine, Suita, Japan; 3 Department of Epidemiology and Public Health, University College London, London, United Kingdom

**Keywords:** socioeconomic inequality in smoking, tobacco control, inverse equity hypothesis, equity effectiveness loop

## Abstract

Previous systematic reviews of population-level tobacco control interventions and their effects on smoking inequality by socioeconomic factors concluded that tobacco taxation reduce smoking inequality by income (although this is not consistent for other socioeconomic factors, such as education). Inconsistent results have been reported for socioeconomic differences, especially for other tobacco control measures, such as smoke-free policies and anti-tobacco media campaigns. To understand smoking inequality itself and to develop strategies to reduce smoking inequality, knowledge of the underlying principles or mechanisms of the inequality over a long time-course may be important. For example, the inverse equity hypothesis recognizes that inequality may evolve in stages. New population-based interventions are initially primarily accessed by the affluent and well-educated, so there is an initial increase in socioeconomic inequality (early stage). These inequalities narrow when the deprived population can access the intervention after the affluent have gained maximum benefit (late stage). Following this hypothesis, all tobacco control measures may have the potential to reduce smoking inequality, if they continue for a long term, covering and reaching all socioeconomic subgroups. Re-evaluation of the impact of the interventions on smoking inequality using a long time-course perspective may lead to a favorable next step in equity effectiveness. Tackling socioeconomic inequality in smoking may be a key public health target for the reduction of inequality in health.

## INTRODUCTION

It is a paradox that, while certain health programs can improve the average value of overall population health, they can actually increase health inequality by socioeconomic status. From an equity perspective, strategies are needed to ensure that an improvement in total health and a reduction in inequality are achieved concurrently. Regarding this issue, we have drawn attention to the impact of tobacco control measures on socioeconomic inequality in smoking. Most tobacco control measures, such as tobacco taxation, smoke-free legislation, and anti-tobacco media campaigns, have proved effective in the reduction of smoking prevalence.^1^ However, there is little evidence about the impact of these measures on smoking inequality.^2^^–^^4^ Therefore, the objective of this article is to provide deep insights into the impact on smoking inequality as an aspect of the effect of tobacco control measures. The scope of this article includes the necessity of these measures, underlying mechanisms, evaluation models, and future implications.

## WHY ARE TOBACCO CONTROL MEASURES TO REDUCE SMOKING INEQUALITY NECESSARY?

Reducing socioeconomic inequalities in health is a priority for public health worldwide, including Japan. The World Health Organization (WHO)’s Commission on Social Determinants of Health recommended monitoring and evaluating socioeconomic inequalities in health and health behavior.^5^ This recommendation is followed in Japan’s new health promotion strategy, Health Japan 21(Second term).^6^ Regarding social inequalities in health, tobacco smoking has been shown to be a major contributor and is the greatest single contributor to preventable death and disease worldwide and in Japan.^7^^–^^9^ Reducing the socioeconomic inequality in smoking may lead to a reduction in health inequality; which in turn may lead to overall health promotion.^5^^,^^10^

The WHO Framework Convention on Tobacco Control is an evidence-based global public health treaty that suggests solutions to tobacco-related problems for countries and governments.^1^^,^^11^ Several tobacco control measures, such as tobacco taxation, smoke-free legislation, and anti-tobacco media campaigns, have been shown to contribute to an improvement in people’s health.^1^ On the other hand, although these measures have also been investigated to determine whether they reduce socioeconomic inequality in smoking,^2^^,^^12^^,^^13^ they have, as yet, yielded inconsistent results. This suggests the need to discuss the context and the underlying mechanisms.

## SOCIOECONOMIC INEQUALITY IN SMOKING

**Figure 1.**
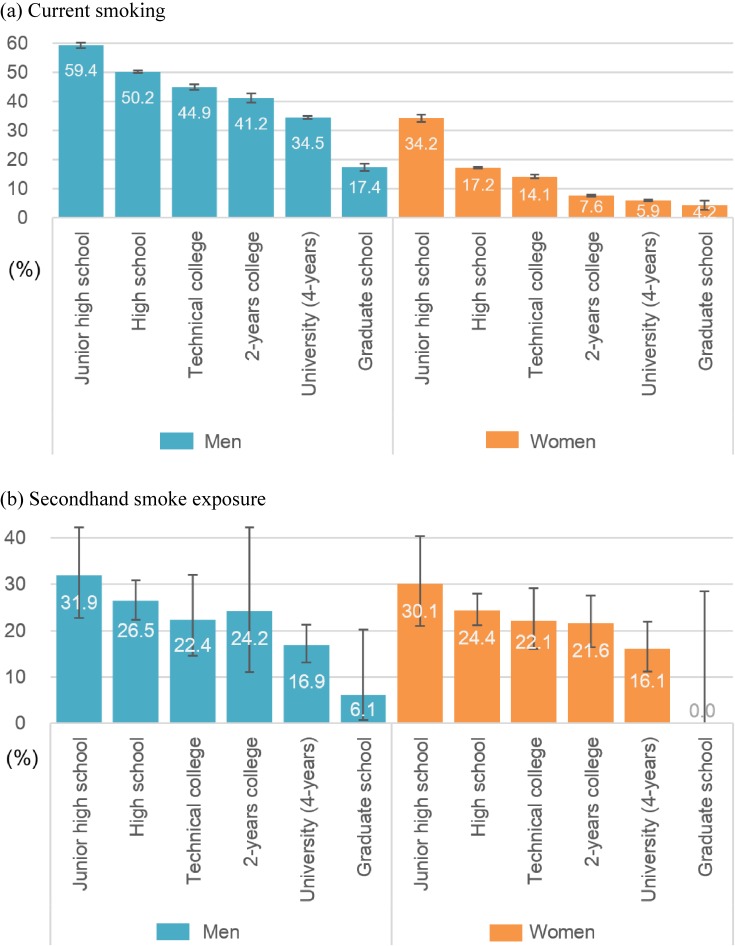
Socioeconomic inequality in smoking in Japan. (a) Education and current smoker prevalence (%). (b) Education and secondhand smoke exposure (%) Note. (a) Percentages of current smoking prevalence (every day or sometimes) among men and women aged 25–64 years (age-adjusted using the direct standardization method) from data from the 2010 Comprehensive Survey of Living Conditions of People on Health and Welfare (CSLC) and the 2010 Japanese Census in Japan.^13^ (b) Percentages of frequent secondhand smoke exposure at home or workplace among men and women aged 25–64 years from linkage data from the 2010 CSLC and the 2010 National Health and Nutritional Survey (NHNS) in Japan.^14^ Because NHNS has much a smaller sample size than CSLC, the ranges of bars (95% confidence intervals) was wider in NHNS.

Smoking inequality has been monitored throughout the world, including Japan. Socio-demographic factors, such as education, income, occupation, gender, ethnicity, and age, have been used as analytical dimensions to estimate smoking inequalities.^2^^,^^9^^,^^12^ For example, educational attainment is a representative socioeconomic factor.^14^ The United States surgeon general’s report showed educational gradients in smoking using four education levels among adults aged 18 years or older^9^: 31.5% (36.2% for men and 26.5% for women) of adults who had education of less than high school smoked currently, compared with 10.4% (11.1% for men and 9.7% for women) of college graduates. Similar patterns were reported in European countries^15^ and Japan.^16^ Among Japanese men aged 25–64 years (Figure 1A), junior high school graduates had the highest current smoking prevalence (59.4%), and graduate school graduates had the lowest (17.4%); high school graduates had the second highest (50.2%). Among women, junior high school graduates had the highest prevalence (34.2%), and graduate school graduates had the lowest (4.2%).^16^

We need to monitor socioeconomic inequality in passive as well as active smoking. Inequality in frequent secondhand smoke exposure (“almost every day”) at home and/or workplace was striking among non-smokers aged 20–69 years old, based on data from two nationally representative cross-sectional studies that were conducted by the Japanese Ministry of Health, Labour and Welfare in 2010 (Figure 1B).^17^ Among both sexes, a high percentage of secondhand smoke exposure was observed in the low education group (30–32% in junior high-school graduates and 24–27% in high-school graduates), and a low percentage of exposure was observed in the high education group (0–6% in graduate school graduates and 16–17% in university graduates).

## IMPACT OF TOBACCO CONTROL MEASURES ON SMOKING INEQUALITY

A previous systematic review (2008)^2^ of population-level tobacco control interventions and their effects on smoking inequalities by socioeconomic factors concluded that tobacco taxation may have the potential to benefit the poor, but yielded mixed results for socioeconomic differences, especially for other tobacco control measures, such as smoke-free policies. The conclusions did not change in the updated review (2014)^3^: ie, increased prices due to tobacco taxation consistently show great potential to reduce smoking inequalities by income. However, this was not true for other socioeconomic factors, such as education. Furthermore, other tobacco control measures, such as smoke-free policy, were assessed as unlikely to reduce inequalities in smoking without specific efforts to reach socioeconomically disadvantaged smokers.^2^^,^^3^ If the smoke-free policy covers all workplaces, it will narrow smoking inequality. However, smoke-free legislation in bars and restaurants is less likely to be enforced in socioeconomically disadvantaged areas.^3^

On the other hand, smoking cessation interventions targeted at socioeconomically disadvantaged groups have also been evaluated, and a systematic review^18^ concluded that smoking cessation interventions (such as brief advice and behavioral support) for socially disadvantaged groups may be effective; however, like the previously mentioned systematic reviews, the overall findings of this review were inconsistent. Thus, further research for both the total population and vulnerable socially disadvantaged groups is necessary.

Until recently, no study had evaluated tobacco control interventions on smoking inequality in Japan. In October 2010, the tobacco tax was increased in Japan, and the tobacco industry simultaneously increased the price for its own benefit. The price of a pack (20 cigarettes) of the most popular brand in Japan, *Mild Seven* (the brand name was changed to *Mevius* in 2013), increased from 300 yen to 410 yen (a 37% increase).^19^ The 2010 tobacco price increase and its effect on cessation has been reported in two studies.^20^^,^^21^ Previous studies (mostly conducted in Western developed countries) generally found that tobacco price increases promoted smoking cessation more among the poor and the young than among the affluent and the old.^2^^,^^3^^,^^22^ However, similar results were not available in Japan.^20^^,^^21^ This might be due to the low tobacco price in Japan, even after the price increase in 2010, according to the affordability index.^10^ Of all the developed countries surveyed for the index, Japan had the most affordable cigarette price in 2009: people only had to work for 11.5 minutes to earn the price of a pack of 20 cigarettes.^10^ Even after the 2010 price increase, this figure only increased to around 16 minutes, whereas in other developed countries, such as Australia, Canada, and the Netherlands, it was 30 minutes.^10^ The affordability of tobacco may indicate an early step of tobacco price intervention, suggesting that we need to consider carefully the underlying theories or mechanisms around tobacco control interventions.

## UNDERLYING MECHANISMS OF SOCIOECONOMIC INEQUALITY IN SMOKING

**Figure 2.**
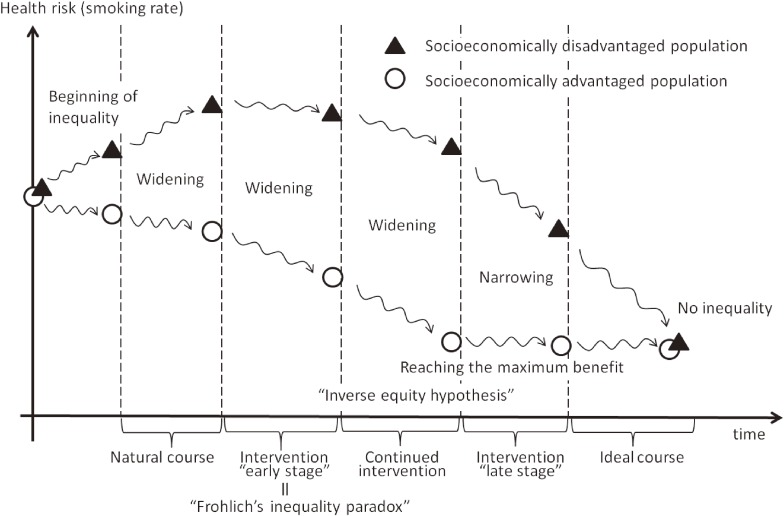
A time course of health inequality based on theories Note: To indicate inequality simply, we illustrated the socioeconomically advantaged and disadvantaged populations.

To understand smoking inequality itself and to develop strategies to reduce smoking inequality, sophisticated knowledge of the underlying mechanisms or principles of trends in inequality is necessary. Figure 2 is a simple representation of the trend of inequality showing the time course of smoking inequality in two groups: socioeconomically disadvantaged and advantaged populations. In the real world, multiple policies will have been implemented at each time stage, but here, for convenience, we assume a situation in which a single policy (which also can be interpreted as integrated multiple policies) was implemented. The time axis usually means yearly units, indicating the several execution phases of that policy.

First, *how does socioeconomic inequality in smoking arise?* Generally, socioeconomically disadvantaged people are more likely to smoke^5^^,^^9^^,^^11^ (corresponding to the phase “beginning of inequality” in Figure 2). According to the Commission on Social Determinants of Health, people embark on a life-course of social disparity as soon as they are born.^5^ Those who experience deprivation in childhood, such as poverty or low educational attainment, are likely to become smokers.^23^ If parents are more disadvantaged socioeconomically, their children are highly likely to suffer secondhand tobacco smoke and more likely to become smokers.^9^ Regarding gender difference in smoking behavior, a typical smoking epidemic pattern within many countries has been observed^11^: first, male smoking prevalence substantially increases, and over the following 3–5 decades, female smoking prevalence increases. However, in most Asian and African countries, including Japan, female smoking has not followed the global epidemic pattern.^11^ Detailed analysis concerning various socioeconomic factors will lead to a better understanding of socioeconomic patterns in the smoking epidemic.

Second, *how does smoking inequality shift over time (with or without tobacco control intervention)?* If critical tobacco control intervention is not carried out (a natural course), the smoking prevalence gap between the affluent (socioeconomically advantaged) and the poor (socioeconomically disadvantaged) may become wider (see Figure 2).^5^^,^^24^ Even if population-level interventions, which seek to reduce smoking prevalence of the entire population, are implemented, these interventions may widen socioeconomic inequalities in smoking,^4^ especially when the benefits are concentrated among the better-off, with little or no benefit to the vulnerable. This early intervention stage of “inequality paradox” was explained by Frohlich et al using the term “the vulnerable population” for the socioeconomically disadvantaged (see Figure 2).^25^ It is increasingly recognized that interventions based on population approaches may lead to unintended exacerbations of health inequalities; therefore, public health strategies that use both entire-population and vulnerable-population approaches to interventions should be considered.^5^^,^^25^^,^^26^ This inequality-sensitive policy development has been called “proportionate universalism”.^26^ As noted above, tobacco price increases have been shown to reduce smoking inequality in many epidemiologic studies.^2^^,^^22^ Therefore, the affordability after the 2010 Japanese tobacco price increase might link to underdevelopment or inadequate proportionality.^20^^,^^21^^,^^26^

Third, *how does smoking inequality shift in a “long-tailed” time-course?* Difference in the intervention effect on smoking inequality may be explained by the inverse equity hypothesis (Figure 2).^12^^,^^27^^–^^29^ New population-based interventions are initially primarily accessed by the socioeconomically advantaged, so there is an initial increase in inequality (early stage). As these inequalities narrow when the socioeconomically disadvantaged catch up (late stage), we may need to consider the stages of tobacco control measures in a long time-course. Although the tobacco price in Japan was increased in 2010, the incremental increase suggests we are still in the “early stage” of the tobacco price intervention. Further price increases may be necessary to alleviate health inequalities and to shift the intervention to the “late stage”. Furthermore, the above studies also identified hard-to-reach populations for tobacco control (ie, groups that are less sensitive to tobacco price increase). Additional tobacco control measures targeting the hard-to-reach subgroups may be required in accordance with the above-mentioned strategy for the vulnerable population.^5^^,^^25^^,^^26^

## WHAT SHOULD WE DO TO REDUCE SMOKING INEQUALITY? EQUITY EFFECTIVENESS LOOP

**Figure 3.**
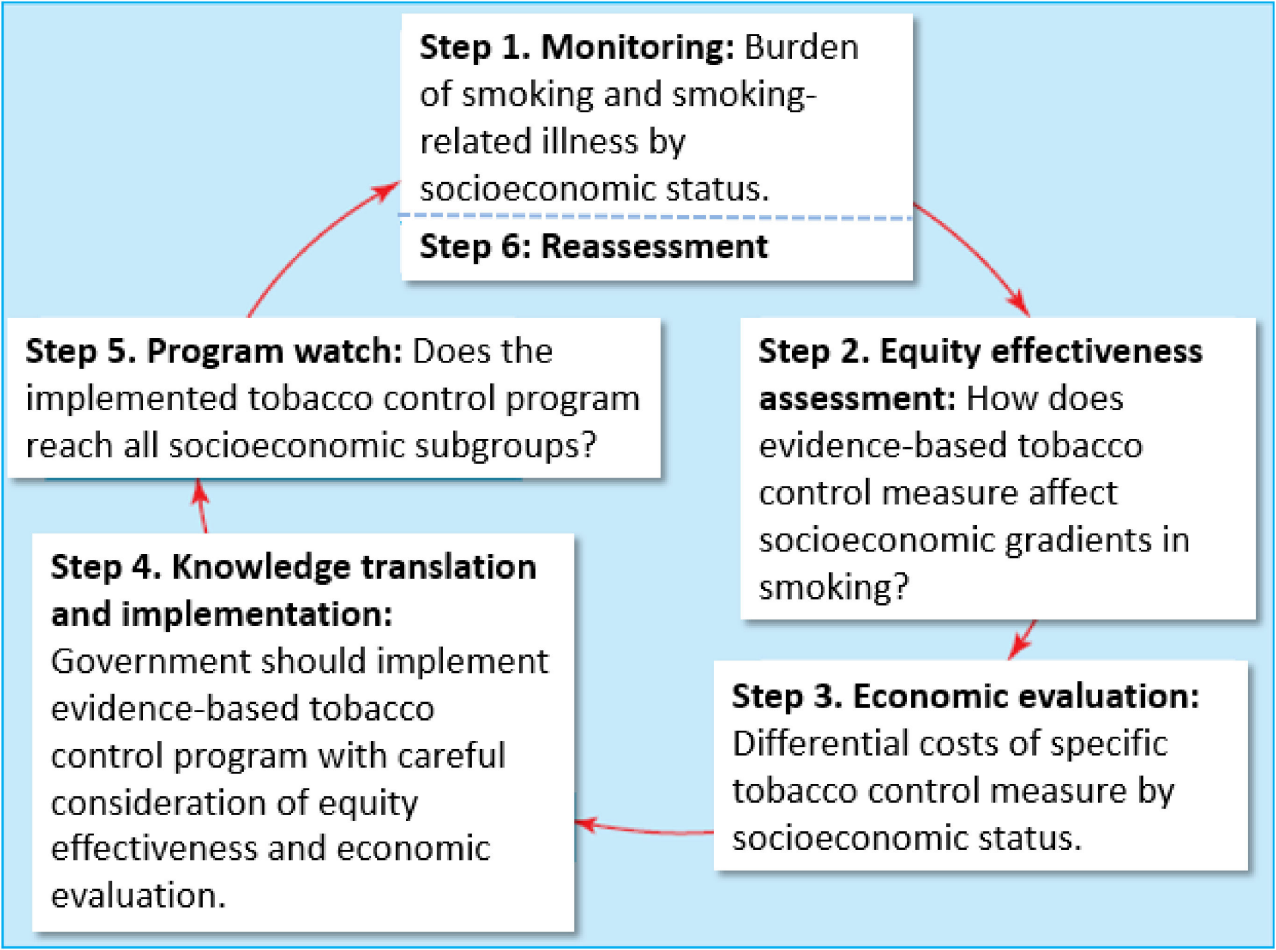
Equity effectiveness loop^*^ for socioeconomic inequality in smoking ^*^We slightly modified the loop by ref. ^30^.

Focusing on the average effects of tobacco control measures may miss important differences within populations. Examining these effects across socioeconomic positions allows the identification of the measures most likely to reduce smoking inequalities. To reduce inequality in health, Tugwell et al suggested an *equity effectiveness loop* that offered a cycle from “monitoring present status” to “effectiveness evaluation and knowledge translation” and then to “re-assessment”.^30^ To evaluate each country’s situation in relation to tobacco control and socioeconomic inequality in smoking, we slightly modified the loop, fitting it to a tobacco control setting (Figure 3). Monitoring and assessment of the present status of tobacco control measures and smoking inequality are the starting points (step 1 and step 2). Economic evaluation of tobacco control by socioeconomic status (step 3) has been conducted, especially focusing on tobacco taxation^9^^,^^22^; however, this has been insufficient in Japan. The tobacco price increase policy, implemented via taxation, has been confirmed to be inexpensive to implement and to have great effect.^1^^,^^9^^,^^22^ Channeling tobacco tax revenues into tobacco control programs is one strategy to make cessation services accessible to the most disadvantaged tobacco users, enabling governments to provide free services to the poor and those without private health insurance.^12^ Such a policy can be seen as an application of “proportionate universalism” designed to reduce overall smoking prevalence and social inequality at the same time. In step 4, tobacco taxation policy should be a priority as an evidence-based tobacco control program to reduce smoking inequality worldwide, including Japan.^1^^,^^31^ However, very few countries—comprising only 10% of the world’s population—had increased tobacco taxes to the best-practice level, defined by WHO MPOWER as more than 75% of the retail price of a pack of cigarettes, by 2014.^1^ Therefore, most countries, including Japan, are evaluated as currently being at step 2 or step 3 of the equity effectiveness loop.

We already know that, even after the 2010 increase, the tobacco price is too low in Japan, as it is in many developing countries.^11^^,^^20^^,^^21^ A previous study in California revealed that the impact of a price increase on purchases only lasted for 4 months after the tobacco price was raised by 95 cents.^32^ Thus, continuous and intensive tobacco price increases are required to promote a reduction in the smoking burden and smoking inequality. After implementing tobacco price increases to the best-practice level (an example of step 4 completion), we need to monitor the effect by socioeconomic status and reassess the burden of socioeconomic inequality in smoking (step 5 and step 6).

## CONCLUSIONS: IMPLICATIONS FOR POLICY AND FUTURE RESEARCH

Previous systematic reviews and other empirical studies have consistently confirmed that tobacco price increase (taxation) reduces smoking inequality by income. A tobacco price increase may be the first priority policy, and there can be little doubt regarding this conclusion. However, previous reviews and empirical studies have not fully evaluated a long-tailed time-course of the effect of tobacco control interventions, suggesting a research gap in the field of tobacco control measures and socioeconomic inequality in smoking. Following the inverse equity hypothesis, continuous and sustained interventions will reduce inequality in the later stages of the policy. In other words (based on the hypothesis), all tobacco control measures may have the potential to reduce smoking inequality. Furthermore, based on the strategy of proportionate universalism, this reduction may be achieved if the measures continue long-term expansion to the best practical level (ie, covering and reaching all socioeconomic subgroups).

Considering the above-mentioned underlying mechanisms, re-evaluation of the impact of the interventions to reduce smoking inequality using a long time-course perspective, which accounts for the inverse equity hypothesis, and of vulnerable or hard-to-reach populations, will be beneficial in future research on health promotion and equity effectiveness. Tackling socioeconomic inequality in smoking may be a key public health target in the reduction of overall inequality in health.
